# Supplementary material for: Are carbon emissions trading and green financial instruments synergistic? -Comprehensive quantitative research based on content analysis
Source: PLoS One. 2024 Mar 7;19(3):e0298601. doi: 10.1371/journal.pone.0298601 (PMC10919601; doi:10.1371/journal.pone.0298601)
Supplement: S1 Data — (ZIP) [file pone.0298601.s001.zip › Data/地级市Z_F工作报告.zip!/║■─╧/│ñ╔│╩╨/└┤╫╘╣1⁄2╓┌║┼í╛┬φ┐╦╩2╛▌═°í┐_2008.docx]

各位代表：

现在，我代表市人民政府向大会作工作报告，请予审议，并请各位政协委员和其他列席人员提出意见。

一、过去五年的工作回顾

　五年来，在市委的正确领导下，市人民政府紧紧依靠全市人民，坚持用科学发展观统领经济社会发展全局，抓住国家实施中部崛起战略机遇，团结奋进，开拓创新，圆满完成了市第十二届人民代表大会第一次会议确定的各项目标任务，全市呈现出蓬勃发展、文明进步、安定和谐的新局面。

　（一）综合实力实现新跨越。2007年，全市地区生产总值达2179亿元（预计数，下同），五年翻了一番，年均增长15．2％，人均生产总值达33000元；社会消费品零售总额达1033亿元，年均增长17．0％；地方财政收入达261．33亿元，是2002年的4．75倍，年均增长36．6％；完成全社会固定资产投资1435亿元，年均增长32．5％；城乡居民人均可支配收入分别达16000元和6316元，年均增长12．1％和13．6％；三次产业比为6．4∶45．0∶48．6，非公有制经济总量占地区生产总值比重达59％。工业总产值达2100亿元，年均增长23．3％；产业集群和支柱产业发展壮大，工业园区集中度显著提高；国企改革和企业办社会职能分离顺利推进。区域性金融中心和芙蓉中央商务区建设全面启动，商贸流通、信息、物流、会展、咨询中介、旅游休闲、房地产业等快速发展。新引进12家世界500强企业，与国内外6个城市缔结为友好城市，实现引进外资银行零的突破。2007年实际利用外资15亿美元，完成进出口总额39．5亿美元，年均分别增长35．4％和18．9％。据国家统计局公布，2006年长沙综合实力居全国城市第14位。在城市综合实力的激烈竞争中位置逐年靠前，五年前进了五位。

　（二）城市建设凸显新风貌。完成“大干新三年、再创新辉煌”城市建设任务，启动实施“再掀新高潮、凸显新风貌”建设工程。完成新一轮城市总体规划修编，实现主城区控规全覆盖。五年投入城市基础设施建设650亿元，新增建成区面积50平方公里，“十纵十横”、“六桥三环”市域路网全部贯通。建设了湘江风光带等一批标志性工程，基本完成背街小巷提质改造和社区环境综合整治。加强生态环境保护和治理，节能减排取得新成效，城市防洪圈和排水截污网基本建成，2007年单位地区生产总值综合能耗降低4％、城市空气优良率达82．5％、城市生活垃圾无害化处理率达100％、全市森林覆盖率达53．6％。探索形成了人性化拆迁、人车分离、集约用地的城建新模式。推进城市管理重心下移和市场化改革，城市管理体制进一步理顺。荣获国家森林城市、全国人居环境范例奖、全国城市管理十佳标兵称号。

　（三）新农村建设呈现新局面。全面取消农业税，对农民实行直补政策。花卉苗木、优质茶叶、优质水产、优质水稻四大优势农业产业带初具规模，动物防疫和农产品质量安全得到保障，农业产业化经营水平有新的提高，2007年实现农产品加工销售收入280亿元。五年投入小城镇建设275．5亿元、投入农村水利44．5亿元。“百村示范”和“千村整治”工程全面实施。新增农村公路5956公里，实现乡乡通油（水泥）路。农村社会事业快速发展，实现了义务教育“一费制”全免、建立了农村低保和新型合作医疗制度，完成一村一卫生室建设。现代农业快速发展，农村面貌焕然一新，农民享受到了更多的发展成果。

（四）文化建设实现新突破。启动创新型城市建设，五年申请专利17733件，连续五年被评为全国科技进步先进市。政府对义务教育的保障作用明显增强，各级各类教育协调发展。加强城乡文化基础设施建设，修缮和保护了12条历史文化街巷，完成简牍博物馆等重大文化项目建设。文艺精品创作成果丰硕，5部作品获全国“五个一工程”奖。被评为全国群众体育工作先进市，两度荣获全国创建文明城市工作先进市。文化体制改革全面推进，文化产业蓬勃发展，原创动漫总量居全国第一。2007年文化产业增加值突破200亿元，约占全市地区生产总值的10％。

（五）社会建设迈上新台阶。加强就业和再就业工作，五年新增城镇就业42.4万人，年均转移农村劳动力72.5万人次，实现城镇无零就业家庭。社会保险费征缴率超过95％，解决4.9万户家庭住房困难，覆盖城乡的社会保障和公共卫生体系基本形成。连续四年实施“八件实事工程”，解决了一批事关人民群众合改革试点市。公共安全、国家安全、食品药品安全、生产安全和普法工作扎实推进。民族团结，宗教和睦。国防教育和民兵预备役建设不断加强。工会、共青团、妇联等群众团体为长沙发展作出新贡献。荣获全国再就业先进集体、全国民族团结进步模范集体、全国社会治安综合治理优秀地市和全国信访工作先进集体，连续四次荣获全国双拥模范城称号。

（六）政府建设取得新成效。深入推进财税体制、投融资体制等改革，设立政府偿债风险准备金，公共财政体系基本建立。推进行政许可集中办理、并联审批和收费公示制，被评为全国政务公开工作先进单位。政府制度建设不断加强，行政决策和运行机制更加规范。自觉接受人大及其常委会的法律监督、工作监督和政协的民主监督。行政监察、行风政风评议、审计执法监督成效明显。办复人大代表建议、批评和意见1078件，政协提案1419件。推进依法行政，五年提请市人大常委会制定、修改和废止地方性法规23件，制定政府规章14件。基层政权建设得到加强，成为全国首批社区建设示范市。统计、人防、台办、市志、档案、新闻出版、公积金管岸、移民开发等工作取得新成绩。

各位代表！过去五年是长沙经济社会快速发展的五年，是人民群众得到实惠更多的五年。这些成绩的取得，是省委、省政府和市委正确领导的结果，是全市人民和衷共济、团结奋斗的结果，离不开市人大、市政协监督和支持，离不开历任老领导、老同志的关心和帮助，特别是第十二届市人民政府领导班子付出了艰辛努力和心血汗水。在此，我代表市人民政府向全市各族人民，向驻长部队、武警官兵和政法，向各民主、工商联、人民团体和各界人士，向所有关心支持长沙发展的国内外朋友，表示诚挚的感谢和崇高的敬意！

回顾过去五年丰富生动的实践，我们有以下深刻启示：一是始终坚持把发展作为第一要务。牢固树立科学发展观，坚持从长沙实际出发，努力探索具有长沙特色的发展道路，确立了兴工强市、科教先导、城乡统筹、文化推动发展战略，做到一切服务于发展，一切服从于发展，用发展解决前进中的困难，用发展检验工作成效，实现又好又快、率先发展。二是始终坚持大力推进城市建设。从城市建设就是经济建设、城市工程就是民生工程、城市投入就是产业投入的高度，全面管理，将历史名城与现代新城建设相结合，以城市建设推进新农村建设，以城市建设塑造城市形象，不断增强城市综合实力和竞争力。三是始终坚持高度关注和改善民生。认真落实发展为了人民、发展依靠人民、发展成果由人民共享的要求，想问题、办事情、作决策，始终尊重群众意愿，充分考虑人民群众的利益诉求，将改革发展成果惠及广大群众。四是始终坚持以长沙精神凝聚发展力量。不断将“心忧天下、敢为人先”的长沙精神注入新的时代内涵，改革和创新经济社会发展的体制机制，营造团结事、和事的浓厚氛围，以卓有成效的发展业绩赢得人民群众的拥护和赞许，凝聚了强大发展合力。

在看到成绩的同时，我们也清醒地看到前进中存在的困难和问题：经济总量还不够大，产业结构需要进一步优化，经济外向度亟待提高；土地紧张和资金短缺等瓶颈制约比较突出；农村基础薄弱，农民持续增收缺乏长效机制；交通堵塞现象比较突出，市容环境仍需改善；节能减排压力加大，治理污染和保护生态需要付出艰巨努力；结构性物价上涨比较明显，低收入群体和特困家庭生活受到一定的影响；社会保障水平不高，部分低收入群体在就业、就学、就医、住房等方面存在较大困难；行政效率和服务质量与人民群众的要求仍有不少差距等。我们必须高度重视这些矛盾和问题，采取有效措施认真加以解决。

二、未来五年面临的形势与任务

　各位代表，未来五年，是全面贯彻落实党的十七大精神，迈向建设全面小康社会的关键时期。全球经济形势稳定，国内宏观经济前景良好，控总量、稳物价、调结构、促平衡的宏观调控政策，将在很大程度上抑制和消除发展中的不积极、不健康因素，经济发展呈现稳健上行的态势。中长沙在内的中部26城市比照实施振兴东北老工业基地和西部大开发的优惠政策，发达地区的产业和资本加速向内地转移，经济要素的跨区域流动更加自由、更加广泛，中部崛起势成必然。国家批准长株潭城市群为“资源节约型和环境友好型社会建设综合配套改革试验区”，为长沙发展打造了金字招牌，将极大地提高长沙在全国经济社会发展布局中的地位，有力地推动我们实现自主改革，走生产发展、生活富裕、生态良好的新型工业化道路。省委省政府实施优势地区优先发展战略，长株潭经济一体化和“3＋5”城市群建设的深度推进，将进一步促进资本、人才、技术等方面的优势资源向长沙聚集，形成更加强劲的区域引擎。

经济规律的作用和政策的引导支持，使长沙成为新一轮发展的前沿阵地，使我们站在了一新的历史起点上。通过多年的发展，我们积累了雄厚的科教优势和明显的人力资源优势，东承长三角、南连珠三角的区位优势凸现，基础设施、基础产业、基础工作得到夯实，多年积聚的潜能开始逐步释放。机遇稍纵即逝，我们一定要倍加珍惜，乘势而上，领会中央精神，创新发展理念，把握发展规律，用足用好政策，不负省委、省政府的重托，不负全市人民的期望，不负历史赋予的重大机遇。通过五年的努力，到2012年，提前达到国家2020年的奋斗目标，率先基本实现全面小康，全市经济实力明显增强，社会发展明显进步，生活质量明显提高，生态环境明显改善，人民生活更加富足。

　经济社会主要目标为：增长13％，突破4000亿元，人均生产总值达元；地方财政收入年均增长15％，突破500亿元；全社会固定资产投资年均增长15％，突破2800亿元；社会消费品零售总额年均增长，突破2000亿元。城乡居民人均可支配收入年均增长10％和12％，分别达25000元和11500元；城镇登记失业率控制在4.5％以内，社会保险覆盖率达95％综合能耗降低20％，主要污染物排放总量减少；人口自然增长率控制在5.5‰以内。

　为实现上述目标，未来五年政府工作的总体思路是：全面贯彻落实党的十七大精神，坚持以人为本、科学发展、率先发展、和谐发展，大力推进经济建设、城市建设、新农村建设、社会建设和政府建设，努力把长沙建设成具有国际视野、人民引以自豪的创业之都、宜居城市、幸福家园！

　为此，我们要通过不懈努力，做大总量、做强产业、做优结构，使实力更强、城乡更美、人民更富——

　建设经济发达、活力迸发的创业之都。经济发展方式深刻转变，节能减排富有成效，新型工业化纵深推进，国际化程度明显提高，综合实力迈向省会城市前列。城市更加开，创业为荣、创业为乐深入人心，人气鼎旺，市场繁荣，成为人们创业兴业的理想选择。

建设环境优美、方便舒适的宜居城市。山水洲城风貌彰显，自然与生态环境良好，城市品位和现代化程度不断提升，城市综合配套功能、人居功能显著增强，成为环境优美、生活方便、富有独特魅力的园林型生态城市和区域性中心城市。

　建设生活富足、文明和谐的幸福家园。城乡居民家庭财产普遍增加，人民过上更加殷实的生活，基本实现学有所教、劳有所得、病有所医、老有所养、住有所居。人们精神文化生活需求得到更好保障，群众安全感、幸福感明显增强，成为生活品质良好、文化教育发达、社会秩序安定、人际关系和谐的安居乐园。

各位代表！面对新的形势和任务，要把美好的发展愿景变为现实，必须坚持以下发展理念：

　——坚持科学发展，率先发展。瞄准国内领先、国际先进水平，坚持生态优先，建设生态文明，集约节约土地资源，自觉追求质量、效益、环境相协调的“好”，努力做到经济总量做大和人民得到更多实惠的“快”，在科学发展中增强竞争力，在率先发展中提升影响力，实现“好中求快，优中求进”。

　——坚持以人为本，改善民生。牢固树立以人为本、民生为重的执政理念，把促进社会公平正义作为政府执政的核心价值，突出公共性和普惠性，把新增财力更多地投向民生领域，投向公益事业，投向困难群体和弱势群体，使改革发展的成果惠及更多的大众，群众利益得到保障，人的尊严得到充分尊重。

　——坚持改革创新，与时俱进。以改革为动力，向创新要活力。加大重点领域和关键环节的改革，创立充满活力、富有效率、更加开放的体制机制，增强城市持续创新能力。以国内发达城市为坐标，树立世界眼光，加强战略思维，转变不适应不符合科学发展观的思想观念，努力形成鼓励创新、鼓励创造、鼓励创业的社会氛围。

　——坚持统筹兼顾，促进和谐。统筹城乡发展、经济社会发展、人与自然协调发展，统筹局部利益和整体利益、当前利益和长远利益。按照共同建设、共同享有的原则，全面推进基本公共服务均等化，营造良好的人文环境，形成全市人民各尽其能、各得其所、和谐共处的新局面。

三、对未来五年政府工作的建议

　围绕上述目标任务，未来五年我们将着力抓好五大领域的工作：

　（一）大力推进经济建设，加快转变经济发展方式。始终坚持发展是第一要务，以产业为支撑，以创新为动力，以创业为源泉，推动经济全面步入科学发展轨道，依靠科技进步、节约挖潜和提质增效，做大经济总量，实现又好又快发展，为建设创业之都打牢物质基础。

　提高自主创新能力。以建设创新型城市为目标，加大自主创新投入和技术创新体系、创新源头建设，全面实施标准化战略。坚持人才是第一资源，加大高层次人才的培养、引进和使用力度，促进人才优势转化为科技优势和竞争优势。打造中国（长沙）科技成果转化交易会品牌，促进科技成果转化和产业化，引导和支持创新要素向企业集聚。加强知识产权保护，突出对原始创新技术、关键核心技术的重点突破和集成创新，提升产业核心竞争力，形成一批中国驰名商标和名牌产品的企业群体，实现由“长沙制造”向“长沙创造”迈进。

推进新型工业化。兴工强市是长沙经济发展的核心战略，新型工业化是长沙经济发展的第一推动力。突出发展高新技术产业，推进信息化与工业化融合。突出发展优势产业集群，实施一批重点配套项目，围绕主机企业进行配套协作，着力打造千亿元产业集群。改造提升传统产业，加快发展民营经济和中小企业，着力解决融资难、用地难等问题。突出工业园区发展，推动政策向园区倾斜、产业向园区集聚，优化园区布局，着力解决园区同质化发展趋向，防止无序竞争，形成错位发展格局，把工业园区建成经济发展的核心增长极，攒足发展后劲，预留发展空间。

　加快发展服务业。服务业是长沙的优势与活力所在。坚持传统服务业与现代服务业并重，生产性服务业与生活性服务业并举。加快传统服务业改造升级，积极推进连锁经营，力争5家以上商贸企业和流通市场进入全国行业百强。突出发展物流、金融、信息、会展、咨询中介等现代服务业，加快区域性金融中心、金霞物流保税中心和芙蓉中央商务区建设，促进现代服务业与新型工业深度融合。发挥文化名城品牌优势和人才资源集聚优势，发展壮大影视传媒、动漫游戏等文化创意产业，提升旅游、娱乐休闲、体育保健等产业发展水平，努力建设区域性现代服务业中心和文化创意中心。

　落实“两型”社会配套措施。“两型”社会建设综合配套改革试验是长沙未来最大的发展机遇。要大胆改革创新，做综合配套改革试验的先行者。坚持资源开发与节约并重、节约优先的方针，制定实施产业和基础设施建设用地、能耗、环保等方面的标准，发展资源节约型产业。土地作为最重要的战略资源，要特别注重集约和节约使用，从制度上、管理上采取有效措施，把单位土地利用强度作为考核经济发展和基础设施建设绩效的重要标准，提高土地利用效益。科学安排建设项目，做到可建可不建的坚决不建，坚决制止超预算建设和重复建设。落实节能减排工作责任制，广泛开展建设节约型城市活动，使节能减排成为全社会的自觉行动。大力发展循环经济，倡导节约、环保、文明的生产方式和消费模式。

提高开放型经济水平。充分利用国内外两种资源和两市场，扩大开放领域，提高开放质量。创新利用外资方式，突出重大产业招商、服务外商、现代物流招商、中央商务区建设招商，加大引进外资金融机构力度，2012年实际利用外资达30亿美元。规范招商引资行为，以优良的经济环境和服务质量吸引内外投资，防止和纠正违法违规竞相优惠行为。注重对内开放，加强区域合作，实现对内对外开放相互促岸管理，促进外贸增长方式转变，调整构，促进加工贸易转型升级，大力发展服务贸易。支持企业在发、生产、等方面开展全球化经营，提高企业国际竞争能力。

　（二）大力推进城市建设，精心打造宜居城市。宜居是城市发展的目标，也是城市最可贵的品质。建设宜居城市必须坚持高起精心建设、精细管理，展现街区整齐、色彩素雅、风格简洁、建筑精致、人与自然和谐的城市新风貌。

　实行规划先导。是城市建设的第一要务。城市要坚持以人为本，把宜居重要内容，努力满足人的全面需求。主动对接长株潭城市群规积极参与“3＋5际快速交通线建设。适时调整和完善城市总望融城、浏宁扩容提质、卫星城镇和中心城镇有序发展，形成结构合理、功能互补、集中集约、整体优化的市域城镇架构。强化在城市建设中的法律地位与权威，严格以引导项目，以指导建设。到2012年，实现建成区面积达300平方公里，城区达300万的区域性现代化中心城市发展目标。

　实施畅通工程。启动和建设黄花机场扩建配套、武广长沙段、霞凝港三期、轨道交通等一批重大交通设施项目，构建立体型、枢纽型、网络型的大交通格局。实施公交优先战略，科学设计和优化公交线路，加强站场建设，扩大公共交通承载能力和覆盖面。优化交通组织结构，建设一批“零换乘”工程，打通城市微循环，实现三环成网、动静结合的市内交通体系，提高运行效率。

　实施品质工程。在城市建设中更多地融入文化内涵与现代先进理念，追求建筑风格与色彩的和谐统一。以橘子洲和湘江风光带，省府新区和市府新区，新河三角洲－五合垸、武广长沙站、岳麓滨江、南湖、湘江明珠五大片区为重点，大胆精心实施，建设经久耐用、美观大方的精品工程，保护和修缮一批历史文化街巷，加快棚户区和城中村改造，不断提高市民的亲切感、认同感和归属感，努力彰显山水洲城、文化名城的特质风采。

实施生态工程。推进生态示范市建设，建立生态补偿机制，建设环境友好型城市。开展清洁能源行动和城市洁美行动，加强水污染、大气污染、土壤污染、生活垃圾污染治理。构建城市林业生态圈和生态绿地系统，提质改造岳麓山风景区，建好10生态主题公园和浏阳河、圭塘河风光带。保护好山体和水系，坚决禁止先推山毁林再造景植树的行为。到2012年，人均公共绿地面积增加2平方米，城市污水处理达标率100％，空气优良率90％以上，使天更蓝、水更清、城更绿、空气更洁净。

　实行精细管理。加强城市综合管理，将管理纳入政府目标考核。在树立全寿命周期成本理念，既考虑当前建设的需要，又满足未来管理的方便，减少后期维护成本，提高城市管理效率。稳步推进城市管理综合执法，提升城市管理科技含量和科学水平，构建城市管理长效机制。加快城市管理市场化步伐，探索推行社会化、物业化管理模式。明晰城市管理责任，实行“区域式联动、网格化管理”，引导市民作为志愿者参与城市管理，充分发挥街道、社区等基层组织作用，让城市管理延伸到每个社区每条街巷。坚持人性化管理，创新和优化管理体制、制度、手段和方式，实现管理高效化、精细化、规范化，为市民创造文明、卫生、整洁、有序的城市环境。

　（三）大力推进新农村建设，实现城乡统筹协调发展。按照“工业反哺农业，城市支持农村”和“多予、少取、放活”的既定方针，始终坚持农业基础地位不动摇，始终坚持加强、支持、保护农业不动摇，始终坚持把解决“三农”问题作为全部工作的重中之重不动摇，努力形成城乡经济社会发展一体化新格局。

　推进城乡规划布局一体化。统筹城乡发展，规划先行。要将城乡作为一个整体统筹规划和科学布局，形成城乡协调的空间结构体系、产业布局体系、基础设施体系、公共服务体系、生态环境体系。认真实施城乡规划法，加快完善市域总体规划、县域总体规划、乡镇和村级规划，实现城乡规划全覆盖。加强城市总体规划与土地利用规划的衔接，实现规划的有机结合，以规划推进新农村建设。

　推进城乡产业发展一体化。坚持把繁荣农村经济，增加农民收入作为新农村建设的中心任务，建立?邹促进农民持续增收的长效机制和产业格局。壮大县域经济，发展都市型、生态型和特色型农业，推进四大农业优势产业带建设，加快长沙新农村示范片建设，提高农业现代化水平。巩固和扶持粮油生产，发展生猪适度规模养殖，抓好“菜篮子”工程。发展农民专业合作组织，促进农村土地有序流转，推进农业产业化经营，构建生产、加工、销售有机结合的高效农业产业体系。增强农业科技创新能力、储备能力和转化能力，建设区域性农产品加工中心、农产品流通中心和农业科教中心。

　推进城乡基础设施建设一体化。加大农村基础设施建设投入，增幅同比提高2个百分点以上，推动城乡基础设施对接联网、互通共享。完成病险水库除险加固，基本解决农村饮水安全问题，完成县乡公路硬化改造，实现广播电视村村通。加强中心城镇和小城镇建设，重点打造20个特色城镇。深入推进“万村千乡”和“双百”市场工程建设，继续推进以“三整三改一分离”为主要内容的百村示范、千村整治工程，加强农村环境保护，改善农村生产、生活、生态环境。

　推进城乡公共服务一体化。公共财政和新增财力重点向农村倾斜。加快发展农村医疗卫生、教育、就业、社会保障等事业，逐步实现城乡基本公共服务均等化。加强技能培训，让更多农民成为有文化、懂技术、会经营的新型农民。建立健全城乡统一就业体系，扩大农村劳务输出。落实支农惠农政策，加强消赤减债，防止乡镇、村新一轮负债。加强基层政权建设，发展基层民主，完善村务公开，实现政府行政管理与基层群众自治的有效衔接和良性互动。

　（四）大力推进社会建设，切实保障和改善民生。要把以人为本最终落实到为人民谋福祉上，更加注重社会建设，更加倾力改善民生，让人民共享改革发展的成果。

　实施素质提升工程。公民素质是城市的重要软资源、软实力。优先发展教育事业，实施素质教育，继续抓好义务教育，加快发展适应产业需求、紧贴市场需要的职业教育、继续教育和远程教育，提高高等教育质量，构建终身学习、全民学习体系，建设学习型城市。推进文化、体育事业发展，构建设施齐全、结构完备、覆盖城乡的公共文化和体育服务体系，推动文化、体育设施向社会公众开放。加强人才工作，建设人力资源强市。稳定低生育水平，提高出生人口素质。建设创业、责任、诚信、和谐文化，构筑全市人民共享的精神家园。

实施创业就业工程。实施积极的就业政策，鼓励支持自主创业、自谋职业，积极培育创业主体，使更多劳动者成为创业者，以创业带动就业。建立就业援助制度，动态消除零就业家庭，帮助解决户籍人口大学生就业难题。加强劳动技能培训，加大农村富余劳动力转移力度。积极推进企业社会责任制度建设，依法维护劳动者权益。深化收入分配制度改革，形成科学合理的收入分配机制。

　实施安居工程。保持房地产业持续稳定健康发展，健全多层次住房保障体系，满足人们住房多样化需求。改进和规范经济适用住房制度，建立健全城市廉租住房制度，五年新建350万平方米经济适用住房、130万平方米廉租住房。改善农村特困群众住房条件，努力做到户户享有一套适宜居住的房屋。

健全基本生活保障体系。完善基本养老保险制度，做实养老保险个人账户，建立农村养老保险和失地农民保障制度，实现基本养老保险城乡全覆盖。完善最低生活保障制度，提高农村五保户集中供养比例。发展老龄事业，建立养老服务体系。加快发展社会救助和慈善事业，完善优抚、扶老、助残、救孤、济困等社会福利制度，为城乡特困群众和弱势群体基本生活提供保障。

　健全基本医疗卫生保障体系。推进健康型城市建设，健全城乡居民公共卫生、医疗服务和药品供应保障体系，从制度上保障人民健康。扩大工伤保险覆盖面，完善城镇居民医疗保险制度、大病医疗救助制度、特殊医疗补助制度，提高农村合作医疗保障标准，加强农村三级卫生服务网络和社区卫生服务中心建设，做到“小病不出村（社区），大病有保障”，为群众提供安全有效、方便价廉的医疗卫生服务。

　健全社会安全保障体系。加强城乡社区建设，使之成为管理有序、服务完善、文明祥和的社会生活共同体。依法管理民族宗教事务，促进民族团结进步，发挥宗教界的积极作用。完善社会管理体制，加强社会工作人才队伍建设，关心基层工作人员。做好信访工作，妥善处理和化解各类矛盾。加强食品、药品和饮水安全监管。推进平安长沙建设，完善突发公共事件应急机制。健全国防教育动员体系，加强国防后备力量建设，继续推进双拥共建。增强国家安全意识。让城乡居民安心、舒心、开心。

　（五）大力推进政府建设，提高政务服务水平。提高公务员队伍素质，建设行为规范、公正透明、勤政高效、清正廉洁的政府，让人民感到我们的政府是真正对人民负责、为人民办实事的政府。

　转变政府职能，建设服务政府。公共服务是政府的主要职能，是现代政府的显著特征。坚持依法行政，改进政府立法工作，完善政府重大决策规则和程序，推进决策科学化、民主化、法制化。牢记为民服务宗旨，强化政府公共服务职能，完善基本公共服务体系。坚持依法理财治税，公共财政重点向民生倾斜，向提高发展能力倾斜，向急需解决的问题倾斜。创新政府管理和服务方式，逐步将服务重心下移到社区和行政村，为城乡居民提供方便快捷的服务。

　提高行政能力，建设效能政府。以提高行政执行力为核心，按照奖优、罚劣、惩庸原则，加强公务员队伍建设，加快建设“政府绩效评估系统”。政府实施重点改革、重大政策、重大工作、重大投入，实行严格的目标管理、严格的督查督办、严格的责任追究，全面提高行政效能。深化行政审批制度改革，简化办事程序。按照中央和省的统一部署，推进行政管理体制改革，探索实行职能有机统一的大部门体制。完善政府投资项目预决算控制和经济责任审计制度，实行跟踪问效。全面推行行政问责制，有责必问，有错必究。

　树立良好政风，建设阳光政府。全面加强政府机关的学风建设和作风建设，加强理论学习，转变思想观念，弘扬新风正气，反对形式主义、官僚主义和铺张浪费。全面推行政务公开，实行城乡规划审批公开，土地供应审批公开，公共资源与资产处置公开，财政预决算报告和审计结果公开，加快政府重大决策向社会公众开放步伐，让政府公共权力在“阳光”下运行，提高政府工作的透明度与公信力。加强电子政务建设，逐步推行网上咨询、网上申报、网上审批、网上许可。依法接受人大法律监督、工作监督和政协民主监督，强化审计监督和行政监察，自觉接受新闻舆论和社会公众监督。贯彻标本兼治、综合治理、惩防并举、注重预防的方针，坚持从严治政，拓展从源头上防治腐败的工作领域，建设为民、务实、清廉的政府。

四、2008年的工作重点

　2008年是全面贯彻落实党的十七大精神的第一年，也是新一届政府的开局之年。全市经济社会发展要立足于稳中求进，好字优先，改革开放，改善民生，推动科学发展，促进社会和谐。主要目标是：地区生产总值增长13％；地方财政收入增长15％以上；全社会固定资产投资增长20％；社会消费品零售总额增长16％；单位地区生产总值综合能耗下降4％，主要污染物排放总量减少4％；城乡居民人均可支配收入分别增长10％和12％以上；城镇登记失业率控制在4．5％以内；人口自然增长率控制在5．2‰以内；居民消费价格指数控制在全国省会城市平均水平以内。

　为此，我们将重点抓好以下几个方面的工作：

　（一）加快新型工业化进程。依托园区实施集聚发展。创新园区管理机制，把园区工作重心转到招商引资和协调服务上来。引导中小企业和民营企业向园区集中，招商引资产业项目向园区集聚。启动经济新区的规划建设，加快园区的扩容提质和升级，提高产业集聚度。依托项目实施集群发展。加快六大优势产业集群发展，积极培育住宅工业、数字媒体、农产品加工、浏阳花炮、服装服饰等产业集群，抓好中联、三一、山河智能、江麓容大、金瑞等重点项目建设，抓好科技成果转化基地、光伏产业基地、化工产业基地等重点项目载体建设。实施中小企业成长工程，鼓励发展“专、精、特、新”项目，延长产业链条。依托创新实施集约发展。重点打造中试、对接、服务和投融资四大平台，突出抓好重点行业、重点领域节能减排工作。鼓励企业争创品牌，全年新增中国名牌产品2个、国家免检产品10个、中国驰名商标2个。

　（二）增强农业和农村发展活力。提升农业产业化经营水平。加快农业优势产业带建设，抓好现代农业“十大标志性工程”，全市农产品加工业产值增长18％以上。发展生态农业和高效农业，重点建设5个市级农业科技园区。实行动物免费强制防疫，建立农产品质量可溯制度和市场准入制度。提高农民组织化程度。深入推进“万村千乡”工程，健全土地承包经营权流转市场，抓好土地整理与开发。大力发展农民专业合作组织，农民参与率达35％以上。开展新型农民科技培训30万人次以上。加强农村基础建设。投入1000万元编制村镇规划，村镇规划覆盖率达50％以上。重点扶持区域带动功能强的中心城镇建设。加大公共财政对农村投入的比重，改善农村生产生活条件，解决9．5万农村人口饮水安全问题，新建沼气池1万个，新建改造农村公路1700公里。继续抓好偏远农村电网改造。开展农村新社区建设试点工作。继续实施“三整三改一分离”，全面开展村庄环境整治。

　（三）加强城市建设与管理。注重提升规划水平。突出做好湘江风光带景观规划和城市建筑风格与色彩规划。以构建大交通、打通微循环、对接长株潭为重点，做好城市交通规划。以城乡统筹，突出特色，提高品位为重点，做好城乡结合部、中心镇的规划。出台和实施城乡规划管理办法，加大规划执行力度。着力提升城市品质。加快省府和市府区域道路、生活服务设施建设，启动五大片区建设和岳麓山风景区提质改造工程，抓好橘子洲提质开发和湘江风光带南北延伸工程。严格控制推山造地建房，重点治理城市噪音、扬尘污染和机动车尾气污染，推动湘江流域环境共保，启动浏阳河、捞刀河、沩水河生态保护，加快株树桥饮水工程建设，完成岳麓、花桥、新开铺污水处理项目续建工程。改造棚户区50万平方米以上，基本完成社区改造达标。启动实施畅通工程。改造中心城区重要交通节点，畅通二环以内城区主干道路网。启动湘江过江通道和城市轨道交通等重大项目建设。加快319国道、长沙大道、学士联络线三个出城口改造。推进国省道及干线公路改造和县际、乡际公路建设，加快黄花机场扩建、武广长沙站、长花灰韶公路、长浏高速、霞凝港三期等重点项目建设，建设金洲大道和南北站迁建配套工程，完成营盘中路改造。优先发展公共交通，优化公交线路，合理布局公交站点，加大站场建设力度，规范公共交通运营秩序。强化城市科学管理。按照“监管分离、市场运作、以块为主、重心下移”原则，完善城市管理体制。实施精细化城市维护作业，努力减少对城市运行的影响。全面落实门前卫生责任制，着力抓好背街小巷、出入城道路、城郊结合部、铁路沿线等薄弱部位市容环境综合整治。广泛开展群众性文明创建活动，重点抓好文明城市示范区和示范行业创建工作，实行文明创建工作责任追究制度，力争2008年成为全国文明城市。

　（四）提升服务型经济发展水平。大力发展生产性服务业。加快芙蓉中央商务区、芙蓉金融街建设。发展产业投资基金，抓好已上市公司的培育，积极推动优质企业上市融资。壮大区域中转物流，发展口岸经济，抓好金霞保税物流中心、霞凝港口岸建设，支持物流骨干企业发展。积极发展知识密集型服务业。发展信息、创意、研发、会展、咨询中介等服务业。构筑服务业信息网络平台，扩大软件、电信、互联网等信息服务规模，大力发展电子商务。支持文化创意产业发展，推进国家动漫游戏产业振兴基地建设，做大做强三辰、宏梦等骨干企业和虹猫、蓝兔等优势品牌。培植和打造会展品牌。优先发展服务外包业，启动全国服务外包基地示范城市建设。着力提升生活性服务业。改造提升五一广场、东塘等中心城区传统商务圈，加快朝阳、红星、中信新城等新型商务区建设。促进房地产业健康稳定发展。发展商贸流通、酒店餐饮、休闲旅游等服务业，促进便利型超市、家政、社区医疗等服务业发展。打造红色旅游、文化旅游和生态旅游品牌。

　（五）扎实推进社会建设。实施积极就业政策。加强创业服务和职业培训，及时帮助失业人员和零就业家庭解决就业困难，全年新增城镇就业岗位8万个以上，确保公益性岗位5000个以上，新增转移农村劳动力6万人。完善社会保障和救助体系。扩大社会保险覆盖面，提高征缴率。企业职工基本养老保险个人账户做实到5％，城镇居民医保、新型农村合作医疗参与率提高到70％和92％。实施新的征地补偿安置条例，妥善解决被征地农民生活生产保障问题。加强市场监测，稳定市场物价，落实因基本生活必需品价格上涨对低收入群众的补助。新建经济适用房70万平方米，廉租房10万平方米。完善困难群体救助制度，适度提高救助标准。加快构建涉老服务体系，全市基本实现敬老院“一乡（镇）一院”建设目标。繁荣社会事业。完成70％以上中小学校的标准化建设，加快职业教育基地建设和明德中学、长郡中学拓址新建。稳步实施医药卫生体制改革，实行乡镇卫生院和社区卫生服务中心临床常用药零差价销售。突出抓好广播电视村村通、乡镇（社区）综合文化站和农家书屋建设，探索网吧长效管理模式。广泛开展全民健身运动。维护安定团结。完善信访制度，提高突发事件应急处置能力。建立健全安全生产长效机制，严防重特大安全事故的发生。加强食品、药品和农产品安全监管，确保群众健康安全。深化“平安长沙”建设，加强社会治安综合治理，保障群众安居乐业，使长沙成为全省的首善之区。

　（六）拓展改革开放的广度和深度。推进和深化各项改革。继续推进行政管理体制改革，探索实行职能有机统一的大部门体制。深化财税、投融资体制改革。整体推进企业办社会职能分离，完善国有资产监管机制。推进事业单位分类改革，探索建立“以费养事”为主体的财政经费供给新机制。落实促进非公有制经济发展政策，优化民营企业发展环境。扎实推进农业保险试点、农村土地征用、农村集体林权制度、兽医管理体制等农村综合配套改革。加强产业招商和项目招商。精心筹备“2008中国·长沙（韩国）现代制造业项目投资洽谈会”、“长沙六大产业集群招商引资推介合作洽谈会”，积极参加第三届中博会、第五届珠洽会等招商活动。突出引进战略投资者，加大总部经济引进、建设力度，实现引进外事、外资机构的突破。拓展对外经贸与区域合作。开拓欧美和非洲外贸市场，培育出口重点企业、出口基地和出口产业集群。支持企业和园区在境外设立产业园，支持优势产业开展境外投资合作。大胆先行先试，加快国家综合配套改革试验区建设，发挥长沙在长株潭经济一体化中的辐射带动作用。加速与“3＋5”城市群的全面合作与产业对接，进一步增进与泛珠三角、中部地区、长三角、东盟等国内外城市间的交流与合作，主动承接产业转移，不断提高经济的开放度和外向度。

　（七）切实加强政风建设。突出抓好政务公开。完善重大决策专家咨询和社会公示、社会听证制度，探索建立决策反馈纠偏机制和决策责任追究制度。市政府常务会议讨论决定与人民群众利益密切相关的重大事项，试行市民代表旁听制度。把与群众利益密切相关的事项和容易滋生腐败的领域作为政务公开的重点，做到审批事项、审批内容、审批结果公开，突出抓好规划、国土审批公开。推行电子监察和网上审批。切实提高服务水平。严格实行首问负责制和一次告知、两次办结制度，落实超时默认、限时办结制度。推行重要岗位内部轮岗和对外交流制度。全面落实行政问责制和行政过错责任追究制度。强化绩效考核，做到奖惩分明，创造优良的投资创业环境。全面落实廉政责任。坚持用制度管权、管事、管人，建立健全决策权、执行权、监督权既相互制约又相互协调的权力结构和运行机制。重点完善国土出让、工程招标、产权交易、政府采购等制度，深入开展商业贿赂专项治理和行风政风评议。厉行节约、勤俭办事，努力用好纳税人的每一分钱，做清正廉洁的表率。

　各位代表！过去的五年，我们谱写了长沙发展史上的新篇章；未来五年，我们肩负着科学发展、率先发展的光荣使命。让我们在市委的正确领导下，紧紧团结和依靠全市人民，为把长沙建设成为具有国际视野、人民引以自豪的创业之都、宜居城市、幸福家园，率先基本实现全面小康而努力奋斗，长沙的明天一定会更加美好！
